# Prevalence of the metabolic syndrome in a rural population in Ghana

**DOI:** 10.1186/1472-6823-12-25

**Published:** 2012-10-30

**Authors:** Mawuli Gyakobo, Albert GB Amoah, De-Anne Martey-Marbell, Rachel C Snow

**Affiliations:** 1 Ghana-Michigan Charter, C/O Office of the Provost, College of Health Sciences, University of Ghana, P. O. Box KB 52, Korle-Bu, Ghana; 2 Department of Medicine and Therapeutics; National Diabetes Management and Research Centre, University of Ghana Medical School, P. O. BOX 4236, Korle-Bu, Ghana; 3 Consultant Family Physician and CEO Mission Clinic, P. O. Box AN 10545, Accra North, West Africa, Ghana; 4 Department of Health Behaviour and Health Education, University of Michigan School of Public Health, 1415 Washington Heights 3814 SPH I, Ann Arbor, Michigan, 48109, USA

**Keywords:** Metabolic syndrome, Rural Ghana, Determinants, ATPIII, IDF

## Abstract

**Background:**

The Metabolic syndrome (MS) which is a constellation of cardiometabolic risk factors including dyslipidaemia, hypertension, hyperglycaemia, central obesity, and endothelial dysfunction was hitherto relatively uncommon among Africans south of the Sahara. This study seeks to determine the prevalence of MS, its components and risk factors among a rural population in Ghana based on two popular international algorithms.

**Methods:**

This was a cross-sectional survey of a rural population in Ghana conducted between November and December, 2007. Two hundred and twenty-eight (228) settler farmers, families and staff associated with the GOPDC Ltd, between the ages of 35 and 64 years, were randomly selected for the study; pregnant women were excluded. The prevalence of MS was estimated using the IDF and ATPIII criteria.

**Results:**

The final subject pool included 102 males, and 104 females. The mean age of all subjects was 44.4 ± 6.9 years. The overall prevalence of MS by the IDF and ATPIII criteria were 35.9% and 15.0%, respectively, but there was an alarming female preponderance by both criteria {IDF: males = 15.7%, females =55.8%; ATPIII: males = 5.9%, females = 24.0%; sex differences p<0.001 for both criteria}. The most important determinants for IDF-defined MS were central obesity (55.3%), low High Density Lipoprotein (42.7%) and high Blood Pressure (39.5%).

**Conclusion:**

The triad of central obesity, high blood pressure and low HDL were most responsible for the syndrome in this rural population.

## Background

Metabolic syndrome (MS) which hitherto was relatively uncommon among Africans south of the Sahara, is increasingly becoming a public health concern in recent times [1]. Several authors have referred to MS by different synonyms prominent among which are Reaven’s Syndrome, Syndrome X, Insulin Resistance Syndrome, Deadly Quartet and Hypertriglyceridemic Waist [2-5]. The syndrome is a constellation of cardiometabolic risk factors including dyslipidaemia, hypertension, hyperglycaemia, central obesity, and endothelial dysfunction [6-10]. MS predisposes individuals to increased risk of cardiovascular disease and type 2 diabetes mellitus and their attendant complications such as heart attack, stroke and renal disease [11,12].

Several criteria have evolved in defining the syndrome. In 1998, the World Health Organisation (WHO) published the first working definition of MS with emphasis on insulin resistance. Subsequently, the European Group for the Study of Insulin Resistance (EGIR) proposed a modification to that of the WHO. The EGIR criterion still required evidence of insulin resistance but added greater focus on abdominal obesity. In 2001, the National Cholesterol Education Programme (NCEP) Adult Treatment Panel III (ATP III) released its definition for MS, de-emphasizing insulin resistance. Subsequently in 2003, the American Association of Clinical Endocrinologists (AACE) modified the NCEP ATP III criterion to refocus on insulin resistance as the primary cause of metabolic risk factors. In the wake of the varied algorithms for MS and consequential confusion in comparing epidemiological studies, the International Diabetic Federation (IDF) in 2005 provided a clinical algorithm for MS that attempts to accommodate the different diagnostic criteria and ethnic differences [5,8,10,13].

High prevalence rates of over 40% have been documented by the IDF criterion in Portugal, Mexico, Urban China, and the United Arab Emirates. India and Brazil also recorded rates above 40% by the NCEP ATP III criterion. Low rates below 10% by both the NCEP ATP III and IDF criteria have been reported in Spain, Japan and Hong Kong [3,6,14], underscoring international variations by both algorithms. Epidemiological studies have reported differences in prevalence rates between males and females in the US [8,15] and Iran [2,11] bringing to the fore the sex sensitivity of both algorithms.

Studies on the MS in sub-Saharan Africa is scanty [1]. Epidemiologic studies in rural Nigeria reported a prevalence rate of 12.1% by the NCEP ATP III criterion but noted similar prevalence rate between males and females [16]. In Ghana, the prevalence and determinants of the MS have not yet been studied in rural communities. This study documents the epidemiology of MS and it’s components in rural Ghanaian males and females, using the NCEP ATP III and IDF international algorithms for MS.

## Methods

### Subjects

This was a cross-sectional survey of a rural population in Ghana conducted from November to December 2007. In arriving at the sample size, an assumed prevalence rate of 50% was used since there was no record of any study on MS in the country. At 95% confidence interval and the degree of accuracy set to 0.05, the desired sample size came to 384. Excluding pregnant women, hypertensives, diabetics, persons not willing to participate in the study and others who were on vacation or could not be traced during the period of the study, the sample size reduced to 228. The subjects included settler farmers, families and staff associated with the Ghana Oil Palm Development Company Limited (GOPDC Ltd), between the ages of 35 and 64 years. Subjects were randomly selected from the staff list in the accounts office and nominal roll in the Human Resource Unit of the Company. Randomisation was facilitated by coded tally cards. Data for 22 subjects out of the 228 were incomplete and discarded as a result of insufficient blood samples.

### Measurements

Two professional nurses were trained on the structured interview guide for the survey. The interview guide was divided into three sections: socio-demographic factors, anthropometric measures and biochemistry.

The interview guide gathered information on demographic factors like age, gender and educational background; risk factors of chronic diseases such as smoking, alcohol intake, diet and physical exercise; prevalence of chronic diseases including hypertension, diabetes mellitus, and dyslipideamia.

The anthropometric measures taken included height, body weight measured in the upright position to the nearest 0.5cm and 0.1kg respectively. The waist circumference (WC) measurements were taken at the end of a normal expiration to the nearest 0.1cm, measuring at the midpoint between the subcostal plane and the supracristal plane.

Mercury sphygmomanometers were used to measure the blood pressure of each subject in the sitting position after 30 minutes of rest. Subjects were asked to refrain from smoking, or ingesting alcohol and caffeine containing products a day before the survey. Three readings each of systolic and diastolic blood pressures were recorded per subject with an interval of five minutes at the least and the mean was used for the data analysis.

### Ethical review and ethics in human subjects research

Ethical approval for this study was given by the Ghana Health Service Ethics Review Committee. Reviewers from the West African College of Physicians have also reviewed the research protocol and granted permission for its execution in fulfilment of requirements towards the award of a Fellowship in Family Medicine. Administrative permission was granted by St. Dominic’s Hospital and the Ghana Oil Palm Development Company where the research was carried out.

All respondents voluntarily participated after the intent and the design of the study had been explained to them and signing informed consent forms prior to implementation of the study.

### Biochemical analysis

Blood sampling was done on the mornings of six Saturdays in the months of November and December 2007 among subjects who had completed the first and second sections of the interview guide. Announcement was made through the community using a public address system the evening preceding blood sampling. Samples were obtained from antecubital veins using 10ml syringes after an overnight fast (10 – 16 hours). Samples for fasting plasma glucose were collected into sodium fluoride/K_3_EDTA bottles and that for fasting lipids were collected into vacutainer serum separated tubes.

Blood samples were immediately analysed for fasting blood glucose using glucometer (“onetouch ultra”) and subjects found to be having abnormal readings were referred to the hospital. Urine samples were also collected and immediately analysed using the 10 parameter test strip. Urine ketones, glucose and proteins were among the parameters under investigation.

The remaining samples were packaged into ice chests and transported to the biochemistry laboratory of St. Dominic’s Hospital (SDH), Akwatia. Samples for fasting glucose and lipid were centrifuged at 5000RPM for 2 minutes and the supernatant plasma and serum respectively collected into plane eppendorf bottles. These were frozen and stored at 4°C.

Samples were collected in duplicate and one set was analysed in SDH whiles the second set was transported in an ice chest to the research laboratory in Korle-Bu Teaching Hospital, Accra within 24 hours for repeat biochemical analysis.

Fasting plasma glucose (FPG) was determined using the enzymatic photometric test according to Barham and Trinder via the “smartlab auto-analyser”. Fasting lipid profile including triglycerides (TG), high density lipoprotein (HDL), low density lipoprotein (LDL), and total cholesterol (TC) were also estimated using the “smartlab auto-analyser”.

TG was measured by the colorimetric enzymatic test using glycerol-3-phosphate-oxidase with the aid of the auto-analyser. HDL was determined after the precipitation of chylomicrons, very low density lipoproteins and low density lipoproteins. Centrifugation left only HDL in the supernatant which was determined enzymatically. Total cholesterol was determined by the enzymatic photometric test. LDL was computed, thus:

$$\;LDL=\text{T}C-\left(HDL+\text{T}G÷5\right)$$

### Diagnostic criteria for metabolic syndrome (MS)

The diagnostic criteria for the MS are listed as follows:

International Diabetes Federation (IDF) criterion:

WC ≥ 94cm in men or ≥80cm in women plus two or more of the following:

a. Low HDL = HDL <40mg/dl in males or <50mg/dl in females, or specific treatment for this lipid abnormality

b. Hypertriglyceridaemia = TG≥150mg/dl, or specific treatment for this lipid abnormality

c. Hypertension = SBP ≥130mmHg or DBP≥85mmHg, or treatment for previously diagnosed hypertension

d. Dysglycaemia = FPG≥100mg/dl, or previously diagnosed type 2 diabetes

National Cholesterol Education Program Adult Treatment Panel (NCEP ATP III) criterion:

At least three of the following criteria:

a. Obesity = WC>102cm in men and 88cm in women

b. Hypertrigylceridaemia = TG≥150mg/dl

c. Low HDL = HDL<40mg/dl in men and <50mg/dl in women

d. Hypertension = BP≥130/85mmHg

e. Dysglycaemia = FPG ≥110mg/dl

### Statistical analysis

Data was analysed using the Statistical Package for Social Sciences, version 15.0 software (SPSS Inc., Chicago, Illinois, USA). Continuous variables were expressed as means and standard deviations and discrete variables were presented as proportions. The prevalence of the metabolic syndrome (MS) among males and females was determined. Age-specific prevalence rates of MS and its components were also determined.

The chi-squares test was performed to test for differences in proportions of categorical variables between two or more groups. In 2*2 tables, the Fisher exact test (2-tailed) replaced the chi-squared test if the assumptions underlying chi-squared were violated, such as situations of small sample size and where the expected frequency is less than 5 in any of the cells.

Multinomial logistic regression analysis was used to determine the type and degree of association between MS and its socio-demographic and behavioural risk factors. The result is presented as odds ratios (ORs) together with their 95% confidence interval (95%CI). The trend in ORs across MS risk factors was evaluated using the likelihood ratio test.

The agreement between IDF and NCEP ATP III criteria of MS was determined by the kappa statistics (k). The level of agreement is considered poor with k≤0.20, fair with k=0.21 to 0.40, moderate with k=0.41 to 0.60, substantial with k=0.61 to 0.80, and very good with k>0.80 [13]. P values of less than 0.05 were considered statistically significant.

## Results

Data on 206 subjects, 102 (49.5%) males and 104 (50.5%) females were analysed. The socio-demographic and behavioural characteristics of the study population by sex are provided in Table 1. There was a significant difference between males and females in terms of age (p<0.001), marital status (p=0.001), family type (p=0.007), educational status (p=0.011), occupation (p<0.001), smoking (p=0.007), alcohol ingestion (p=0.001) and body mass index (p<0.001). No significant difference was observed between the sexes for insurance status. There was a higher prevalence of younger subjects (52.0%) and the females constituted the majority (65.0%). The population was almost universally married (91.3%), especially among males (98.0%). The family type was skewed towards nuclear family (86.8%), and especially among males (93.1%). Nearly 94.0% of the subjects had some education.

**Table 1:** Socio-demographic and behavioural characteristics of the study population by sex

**Variable**	**Male (%)**^**a**^	**Female (%)**^**a**^	**Total (%)**^**a**^	**p**
***Age (Years)***				**P<0.001**
35 – 44	39 (38.6)	67 (65.0)	106 (52.0)	
45 – 64	62 (61.4)	36 (35.0)	98 (48.0)	
Total	101 (100.0)	103 (100.0)	204 (100.0)	
***Marital Status***				***P=0.001***
Single^b^	2 (2.0)	16 (15.4)	18 (8.7)	
Married	100 (98.0)	88 (84.6)	188 (91.3)	
Total	102 (100.0)	104 (100.0)	206 (100.0)	
***Family Type***				***P=0.007***
Open Parent	1 (1.0)	12 (11.7)	13 (6.4)	
Nuclear Family	94 (93.1)	83 (80.6)	177 (86.8)	
Extended Family	6 (5.9)	6 (5.8)	12 (5.9)	
Joint Family	0 (0.0)	2 (1.9)	2 (1.0)	
Total	101 (100.0)	103 (100.0)	204 (100.0)	
***Educational Status***				***P=0.011***
No Education	2 (2.0)	11 (10.6)	13 (6.3)	
Some Education ^c^	100 (98.0)	93 (89.4)	193 (93.7)	
Total	102 (100.0)	104 (100.0)	206 (100.0)	
***Insurance Status***				***P=0.662***
Insured	84 (82.4)	88 (84.6)	172 (83.5)	
Uninsured	18 (17.6)	16 (15.4)	34 (16.5)	
Total	102 (100.0)	104 (100.0)	206 (100.0)	
***Occupation***				**p<0.001**
Sedentary Office Worker	19 (18.6)	1 (1.0)	20 (9.7)	
Active Office Worker	44 (43.1)	70 (67.3)	114 (55.3)	
Field Worker	39 (38.2)	33 (31.7)	72 (35.0)	
Total	102 (100.0)	104 (100.0)	206 (100.0)	
***Smoking***				***p=0.007***
Ever Smoker	15 (14.7)	4 (3.8)	19 (9.2)	
Never Smoker	87 (85.3)	100 (96.2)	187 (90.8)	
Total	102 (100.0)	104 (100.0)	206 (100.0)	
***Alcohol***				***p=0.001***
Ever Drinker	68 (67.3)	45 (43.3)	113 (55.1)	
Never Drinker	33 (32.7)	59 (56.7)	92 (44.9)	
Total	101 (100.0)	104 (100.0)	205 (100.0)	
***Body Mass Index (BMI/kgm***^***-2***^***)***^***d***^				***p<0.001***
< 25.00	72 (70.6)	46 (45.1)	118 (57.8)	
25.00 – 29.99	29 (28.4)	27 (26.5)	56 (27.5)	
≥ 30.00	1 (1.0)	29 (28.4)	30 (14.7)	
Total	102 (100.0)	102 (100.0)	204 (100.0)	

p-values (2-tailed): Expresses the degree of significance between the sexes for each category.

^a^ Percentage in a column.

^b^ Single: Never married + Ever married (Separated, Divorced, Widow, Widower).

^c^ Some Education: Basic + Secondary + Tertiary.

^d^ Normal weight, BMI= <25.00kgm-2; Overweight, BMI= 25.00 – 29.99; Obese, BMI= ≥ 30.00.

Active office workers (Junior staff) were the most prevalent, 55.3% among the study subjects and there was a female preponderance (67.3%). Smoking was rare, at 9.2%, and alcohol consumption was 55.1% prevalent overall with a high male prevalence (67.3%). More than half (57.8%) of the study subjects had normal weight; for males this was true for 70.6%. The obese constituted less than a fifth of the population, and 28.4% of females.

The basic characteristic of the study subjects by sex are shown in Table 2. The mean age, BMI, and WC were 44.40 ± 6.87 years, 25.15 ± 4.67kgm^-2^ and 89.94 ± 10.41cm respectively. The females were younger (42.80 ± 6.18 years) and had higher values of BMI and WC (26.81 ± 5.30 kgm^-2^ and 92.47 ± 11.30cm respectively), and thus were more prone to overweight and obesity. The differences in the means between males and females by selected determinants (age, body mass index, systolic blood pressure, diastolic blood pressure, fasting plasma glucose, high density lipoprotein, triglycerides) of MS were not significant.

**Table 2:** Basic characteristics of study subjects by sex

**Variables**	**Mean ± Standard Deviation**			
	**All (n=206)**	**Males (n=102)**	**Females (n=104)**	**p-values**
Age (years)	44.40 ± 6.87	46.04 ± 7.17	42.80 ± 6.18	0.286
BMI (kgm^-2^)	25.15 ± 4.67	23.47 ± 3.15	26.81 ± 5.30	0.200
WC (cm)	89.94 ± 10.41	87.36 ± 8.75	92.47 ± 11.30	0.225
SBP (mmHg)	125.29 ± 16.97	124.66 ± 13.86	125.91 ± 19.59	0.554
DBP (mmHg)	83.35 ± 14.22	83.38 ± 12.29	83.32 ± 15.94	0.086
FPG (mg/dl)	81.01 ± 22.06	82.73 ± 26.02	79.41 ± 17.60	0.799
HDL (mg/dl)	50.47 ± 17.85	49.86 ± 16.53	51.05 ± 19.06	0.699
TG (mg/dl)	100.38 ± 43.81	97.32 ± 42.40	103.26 ± 45.13	0.176

p-values (2-tailed): Students T-test comparing the mean values between males and females of selected determinants of metabolic syndrome (MS); BMI: body mass index; WC: waist circumference; SBP: systolic blood pressure; DBP: diastolic blood pressure; FPG: fasting plasma glucose; HDL: high density lipoprotein; TG: triglycerides.

Table 3 shows the prevalence of the MS and its determinants by sex as defined by the IDF and NCEP ATP III criteria. The global prevalence of the syndrome was 15.0% and 35.9% by the NCEP ATP III and IDF criteria, respectively. The prevalence by the IDF criterion was more than twice that of the NCEP ATP III criterion. There was a female preponderance in both criteria {IDF (male: female ratio= 1 : 3.6); NCEP ATP III (male : female ratio= 1 : 4.1)} and this trend was significant in both criteria (p<0.001). The kappa statistics for the agreement between the IDF and NCEP ATPIII criteria was moderate {k= 0.446, p<0.001; (results not shown)}.

**Table 3:** Prevalence of the metabolic syndrome (MS) and its determinants by sex as defined by the IDF and NCEP ATP III criteria

**Determinants of MS**	**Prevalence of metabolic syndrome components (%)**							
	**IDF**				**NCEP ATP III**			
	**All (206)**	**Male (102)**	**Female (104)**	**p**	**All (206)**	**Male (102)**	**Female (104)**	**p-values**
Central Obesity	55.3	22.5	87.5	<0.001	31.1	3.9	57.7	<0.001
High BP	39.5	39.0	40.0	0.896	39.5	39.0	40.0	0.896
High FPG	12.1	11.8	12.5	0.872	5.3	5.9	4.8	0.732
Low HDL-C	42.7	32.3	52.5	0.005	42.7	32.3	52.5	0.005
High TG	10.4	9.7	11.1	0.745	10.4	9.7	11.1	0.745
MS	35.9	15.7	55.8	<0.001	15.0	5.9	24.0	<0.001

p- Values (2-tailed): comparing males and females; Central Obesity= IDF {WC≥94cm in males, ≥80cm in females} and NCEP ATPIII {WC>102cm in males, >88cm in females}.

High BP= IDF and NCEP ATPIII {SBP≥130mmHg, DBP≥85mmHg}.

High FPG= IDF {FPG≥100mg/dl}, NCEP ATPIII {FPG≥110mg/dl}.

Low HDL-C= IDF and NCEP ATPIII {HDL <40mg/dl in males, <50mg/dl in females}.

High TG= IDF and NCEP ATPIII {TG≥150mg/dl}.

MS= IDF (International Diabetes Federation) {central obesity and at least two of the following: low HDL-C (or treatment for low HDL-C), high TG (or treatment for high TG), high BP (or treatment for high BP) and high FPG (or treatment for high FPG)}.

MS= NCEP ATP III (National Cholesterol Education Program Adult Treatment Panel III) { at least three of the following: central obesity, high TG, low HDL-C, high BP and high FPG}.

Central obesity (55.3%) was the most prevalent determinant of MS by the IDF criterion followed by low HDL (42.7%) and then high BP (39.5%). The NCEP ATP III criterion showed HDL (42.7%) as the most prevalent followed by high BP (39.5%) and central obesity (31.1%). The sex difference in prevalence rates among the determinants was only significant for central obesity and low HDL by both criteria (p<0.001, p= 0.005 respectively by both criteria). In both the IDF and NCEP ATP III, central obesity in women and high BP in men were the most common determinants and the triad of central obesity, low HDL and high BP constituted the most widespread combination of metabolic abnormalities determining MS.

Table 4 presents the results of age-specific prevalence of MS and its determinants by the IDF and NCEP ATP III criteria. Within the male population, MS did not significantly increase with age by either the IDF and NCEP ATP III criteria (p= 0.679, p= 0.665, respectively). The contrary was observed among the females by the IDF criterion, for whom MS did increase with age (p=0.012). High BP demonstrated significant increase in the trend of MS with age for all three categories; all (p<0.001), male (0.047), female (p<0.001) by both criteria. A similar trend was seen in High TG except in the female group (all: p=0.028, male: p=0.011, female: p=0.380 for both criteria). On the contrary, prevalence of MS significantly decreased with age among the males for High FPG (p=0.020).

**Table 4:** Age-specific prevalence of metabolic syndrome (MS) and its determinants by the IDF and NCEP ATPIII criteria

**MS and Determinants**	**Prevalence of determinants of metabolic syndrome by age and sex (%)**^**a**^					
	**IDF**			**NCEP ATP III**		
	**All**	**Male**	**Female**	**All**	**Male**	**Female**
**MS**						
35-44	34.0	12.8	46.3	14.2	2.6	20.9
45-64	36.7	16.1	72.2	16.3	8.1	30.6
P value ^b^	0.679	0.649	0.012	0.665	0.255	0.276
**Central Obesity**						
35-44	61.3	17.9	86.6	35.8	2.6	55.2
45-64	48.0	24.2	88.9	25.5	4.8	61.1
P value ^b^	0.055	0.459	0.735	0.110	0.568	0.565
**High BP**						
35-44	25.6	25.0	25.9	25.6	25.0	25.9
45-64	55.0	46.9	67.7	55.0	46.9	67.7
P value ^b^	<0.001	0.047	<0.001	<0.001	0.047	<0.001
**High FPG**						
35-44	12.3	12.8	11.9	7.5	12.8	4.5
45-64	12.2	11.3	13.9	3.1	1.6	5.6
P value ^b^	0.997	0.817	0.776	0.156	0.020	0.808
**Low HDL**						
35-44	45.5	38.9	49.2	45.5	38.9	49.2
45-64	39.3	28.6	57.6	39.3	28.6	57.6
P value ^b^	0.387	0.303	0.435	0.387	0.303	0.435
**High TG**						
35-44	5.9	0.0	9.2	5.9	0.0	9.2
45-64	15.7	16.1	15.2	15.7	16.1	15.2
P value ^b^	0.028	0.011	0.380	0.028	0.011	0.380

^a^ Percentage of subjects in that age group out of studied subjects in the same age group.

^b^ p-values (2-tailed) apply to the prevalence of the determinants of the metabolic syndrome across age groups.

The predictive odds of MS (IDF criterion) with variations in socio-demographic and behavioural risk factors are shown in Table 5. The younger age group of 35–44 years were 70.2% (95% CI= 0.125-0.714) less likely to develop MS with reference to the older subjects (45–64 years), while males were 94.0% (95% CI= 0.020-0.178) less likely to develop the syndrome compared to females. The obese and overweight subjects were more than 5, and 10 times, respectively {(95% CI: obese (1.780-17.069), overweight (4.170-26.782)} more likely to develop MS than normal weight subjects.

**Table 5:** Odds ratios of MS according to socio-demographic and behavioural risk factors; applying the IDF criterion

**Variables**	**N (%)**	**p-value**	**OR**	**95% CI**
***Age (years)***				
35 – 44	103 (51.8)	0.007	0.298	0.125 – 0.714
45 – 64	96 (48.2)	Referent	1.0	Referent
***Sex***				
Male	99 (49.7)	<0.001	0.060	0.020 – 0.178
Female	100 (50.3)	Referent	1.0	Referent
***Marital Status***				
Married	181 (91.0)	0.698	0.598	0.045 – 8.000
Single	18 (9.0)	Referent	1.0	Referent
***Family Type***				
Joint Family	2 (1.0)	0.504	3.786	0.076 – 188.597
Open Parent	13 (6.5)	0.556	0.424	0.024 – 7.381
Extended Family	10 (5.0)	0.835	0.830	0.144 – 4.782
Nuclear Family	174 (87.4)	Referent	1.0	Referent
***Educational Status***				
Some Education	189 (95.0)	0.289	2.532	0.454 – 14.124
No Education	10 (5.0)	Referent	1.0	Referent
***Insurance Status***				
Uninsured	34 (17.1)	0.607	0.724	0.211 – 2.481
Insured	165 (82.9)	Referent	1.0	Referent
***Occupation***				
Active Office Worker	109 (54.8)	0.816	0.897	0.360 – 2.236
Sedentary Office Worker	20 (10.1)	0.118	3.270	0.739 – 14.470
Field Worker	70 (35.2)	Referent	1.0	Referent
***Smoking***				
Ever Smoked	17 (8.5)	0.649	0.701	0.152 – 3.241
Never Smoked	182 (91.5)	Referent	1.0	Referent
***Alcohol***				
Ever Drinker	111 (55.8)	0.670	0.840	0.377 – 1.871
Never Drinker	88 (44.2)	Referent	1.0	Referent
***Body Mass Index (kgm***^***-2***^***)***				
Obese (≥ 30)	28 (14.1)	0.003	5.512	1.780 – 17.069
Overweight (25.00 - 29.99)	55 (27.6)	<0.001	10.568	4.170 – 26.782
Normal Weight (< 25.00)	116 (58.3)	Referent	1.0	Referent

## Discussion

This study was conducted among staff, relatives and settler farmers associated with the GOPDC Ltd and thus the findings cannot be generalized to all rural communities in Ghana. In spite of this, these findings are very important to the documentation of metabolic syndrome in modern Ghana.

The prevalence of MS was alarmingly high {35.9% (IDF) and 15.0% (NCEP ATP III)} in this rural community of mainly farmers and small scale industrialist (oil palm extraction). Earlier studies in Cameroon [17], and Nigeria [18], both in West Africa, found much lower prevalence rates. However, a study by Adegoke and fellow researchers in Nigeria determined a rate of 12.7% (NCEP ATP III) that was much close to findings in our study [16]. Studies in the US [8,12,15] and Europe [19-25] revealed higher rates but for a few [26-28] which produced similar findings. This high prevalence rate in Ghana cannot so easily be attributed to a presumed westernization of diet and lifestyle, as these data emerge from a rural, agrarian community with traditional reliance on home-cooked foods, and a high burden of physical labour. Stress could be a major player in this circumstance by triggering a chain of neuroendocrine events culminating in disturbed metabolism. Undocumented environmental exposures or genetic factors may be of research interest in the future.

The IDF criterion yielded a higher prevalence rate (2.4 times) than the NCEP ATP III and this may be due to the lower cut-off points for WC and FPG. Similar trends have been observed in other studies [6,8,27,29,30] but the contrary is also been documented [11,14].

In spite of the differences observed in the prevalence rates by the IDF and NCEP ATP III criteria, the agreement between the two algorithms was moderate, 0.45 as alluded to by Choi and co-workers [31]. Several other studies yielded substantial to very good agreement between the two algorithms [11,13,25,26,32].

There was a significant sex difference in the prevalence of MS in this population. The prevalence was approximately four times higher among females by both algorithms and this is in consonance with other studies with similar, though relatively less dramatic, sex differentials [11,26,31]. The inverse was found by Alegria and fellow researchers [33]. This difference may be due to the significant and higher prevalence of overweight and obese females (55.0%) than males (29.0%) in our study, but more attention is warranted given the likely negative public health implications of these rates among females.

The triad of low HDL, high BP, and central obesity were largely responsible for MS in this community. This combination was also observed in Nigeria [18] and the US [12]. High BP and central obesity were the most prevalent determinants among males and females respectively and this is corroborated by studies in Sweden [28], the US [12], the Hong Kong, Chinese and Hungarian populations [30,34]. This development probably predicts how the two sexes cope with stress.

The prevalence of MS increased with age [6,19] but reached the level of significance only for females (IDF). This trend is supported by findings in the regression analysis where the younger age group (35–44 years) was 70% less likely to develop MS compared to the older group (45–64 years) and age effects could be due to the significant and positive correlation between SBP and DBP with age (results not shown). Hypertriglyceridaemia was also significantly increased with age except for the females and this may be partly due to the positive and significant correlation between age and triglycerides (results not shown). In consonance with the expected, “sedentary office workers” were more than thrice likely to develop MS with reference to “field workers” whiles the contrary was observed for “active office workers” but these findings did not reach the level of significance. Additionally, subjects with “some education” were more than twice likely to develop MS than subjects with “no education” and this also did not reach the level of significance.

## Conclusion

In conclusion, the prevalence of an MS diagnosis was alarmingly high among this population of women in rural Ghana, despite high physical workloads. Consistent with experience elsewhere, the IDF algorithm identified twice as many subjects as having MS, compared to the NCEP ATP III algorithm. The triad of central obesity, high blood pressure and low HDL were most responsible for the syndrome in this rural population. Younger age, male sex, and normal weight were protective against MS. This study needs to be extended to other parts of the country to ascertain the national prevalence rate vis-à-vis, its rural–urban distribution. There is the additional need to determine whether these diagnostic standards are indeed associated with poor long-term clinical outcomes as they are in other parts of the world.

## Abbreviations

MS: Metabolic syndrome; WHO: World health organisation; EGIR: European group for the study of insulin resistance; NCEP: National cholesterol education programme; ATP III: Adult treatment panel III; AACE: American association of clinical endocrinologists; IDF: International diabetic federation; GOPDC: Ghana oil palm development company limited; WC: Waist circumference; SDH: St. Dominic’s hospital; FPG: Fasting plasma glucose; TG: Triglycerides; HDL: High density lipoprotein; LDL: Low density lipoprotein.

## Competing interests

No competing interests were declared by the authors.

## Authors’ contributions

MKG conceptualised and executed the research and was the leading author of the manuscript. AGBA was part of the conceptualisation process and was instrumental in the analysis of the laboratory specimen and writing of the manuscript. DM also played a major part in conceptualising the research and was actively involved in the entire process from data collection through to manuscript production. RCS played a major part in data analysis and manuscript production. All authors read and approved the final manuscript.

## Pre-publication history

The pre-publication history for this paper can be accessed here:

http://www.biomedcentral.com/1472-6823/12/25/prepub
